# Reply: Peripherin is a biomarker of axonal damage in Guillain-Barré syndrome: a pathophysiological annotation

**DOI:** 10.1093/brain/awad276

**Published:** 2023-08-30

**Authors:** Stephen Keddie, Duncan Smyth, Ryan Y S Keh, Luuk Wieske, Milou Michael, Filip Eftimov, Roberto Bellanti, Simon Rinaldi, Axel Petzold, Michael P Lunn

**Affiliations:** Department of Neuromuscular Diseases, Barts Health NHS Trust, London E1 1BB, UK; Department of Neuromuscular Diseases, University College London, London WC1N 3BG, UK; Centre for Neuromuscular Disease, National Hospital for Neurology and Neurosurgery, London WC1N 3BG, UK; Department of Neuromuscular Diseases, University College London, London WC1N 3BG, UK; Centre for Neuromuscular Disease, National Hospital for Neurology and Neurosurgery, London WC1N 3BG, UK; Department of Neuromuscular Diseases, University College London, London WC1N 3BG, UK; Centre for Neuromuscular Disease, National Hospital for Neurology and Neurosurgery, London WC1N 3BG, UK; Department of Neurology and Neurophysiology, Amsterdam Neuroscience, Amsterdam UMC, Location AMC, University of Amsterdam, 1081 HV Amsterdam, The Netherlands; Department of Neurology and Neurophysiology, Amsterdam Neuroscience, Amsterdam UMC, Location AMC, University of Amsterdam, 1081 HV Amsterdam, The Netherlands; Department of Neurology and Neurophysiology, Amsterdam Neuroscience, Amsterdam UMC, Location AMC, University of Amsterdam, 1081 HV Amsterdam, The Netherlands; Nuffield Department of Clinical Neurosciences, University of Oxford, Oxford OX3 9DU, UK; Nuffield Department of Clinical Neurosciences, University of Oxford, Oxford OX3 9DU, UK; Department of Neurology and Neurophysiology, Amsterdam Neuroscience, Amsterdam UMC, Location AMC, University of Amsterdam, 1081 HV Amsterdam, The Netherlands; UCL Clinical and Movement Neurosciences Department, National Hospital for Neurology and Neurosurgery, UCL Institute of Neurology, London WC1E 6BT, UK; Department of Neuromuscular Diseases, University College London, London WC1N 3BG, UK; Centre for Neuromuscular Disease, National Hospital for Neurology and Neurosurgery, London WC1N 3BG, UK; NHS Neuroimmunology and CSF Laboratory, Queen Square Institute of Neurology, London WC1N 3BG, UK

We are grateful to Dr Berciano for his exposition on the pathogenesis of Guillain-Barré syndrome (GBS),^1^ drawing together our findings on the rise in peripherin with a short and early time course and the important historical, clinical and animal model-based literature. These important seminal papers, some from more than 70 years ago, continue to anchor our developments in understanding this disease. Modern data can justify or add to these papers.

The neurophysiological classification of GBS that developed from the work of Feasby, McKhann, Griffin, Ho and others^2–4^ divided GBS largely according to conduction velocities, where reductions in velocity have been considered the direct surrogate of myelin damage or ‘demyelination’. The concept of a spectrum of nodo-paranodopathies, where immunological attack at or around the nodes of Ranvier can result in acute conduction block, paranodal disruption and demyelination or axonal transection, reunified the divergent splitting based on surrogate neurophysiology.^5^ Most recently, work by Cunningham and colleagues^6^ elegantly showed how anti-ganglioside antibody-directed complement-mediated damage results in either axonal or glial calpain activation and cellular damage in and around the node of Ranvier and paranode. Furthermore, axonal nanoruptures result from glial disruption at the paranode, calcium influx and secondary axonal degeneration within hours of the induction of ‘demyelinating’ GBS.^7^ Both of these mechanisms would be expected to result in some loss of axonal integrity and leak of axonal contents.

The pathology in the beautiful papers by Haymaker and Kernohan^8^ and McKhann *et al*.^4^ on the Chinese AMAN cohort came from severely affected GBS cases at autopsy, resulting in strikingly severe and dramatic pathology. Likewise, in P2 passive transfer experimental allergic neuritis (EAN), the phenotypes and pathology can be very variable from mild to very severe where endoneurial fluid pressures (EFP) are increased.^9^ It is entirely feasible that ischaemic injury in the endoneurium exists under these conditions. Increases in intracellular calcium (in minutes to hours), calpain activation, cytoskeletal degradation and cellular disruption could all contribute to swelling and increases in EFP, especially at sites of relative blood–nerve barrier deficiency (spinal roots and distal terminals). But it is also entirely conceivable that cytoskeletal contents can be released through complement-mediated molecular membrane disruption and calpain activation without the swelling and ischaemia seen in the severest of cases. Indeed in the *in vitro* myelinating culture model (see Fig. 2B and D in Keddie *et al*.^10^), visible complement-dependent axonal disruption, and also measurable release of peripherin, occurs within minutes to hours of induction without any requirement for endoneurial restriction and EFP increases. Furthermore, more modest increases in both neurofilament light and peripherin are seen following antibody-mediated complement-mediated demyelination in this model at 4 and 24 h (see Fig. 2C and D in Keddie *et al*.^10^) in the absence of morphological evidence of axonal degeneration at these relatively early time points.

Ongoing work on peripherin is exploring changes in a far larger cohort of deeply phenotyped cases with comprehensive clinical and neurophysiological characterization, and this will clarify whether there are correlations between peripherin levels and causation, outcomes, neurophysiological classifications or other reasonable phenotypic divisions. Whilst we certainly acknowledge that endoneurial ischaemia might contribute to damage and outcomes in GBS, we do not think that our data support any one model for how this cytoskeletal biomarker might be released, particularly with the most recent immunological molecular damage causing disruptions in hours from induction. We remain within touching distance of understanding the pathogenesis of GBS but remarkably remote from the complete picture.

## Data Availability

Data availability is not applicable to this article as no new data were created or analysed in this study.
